# Surgical Removal of an Extrauterine Device Migrating to Appendix

**DOI:** 10.1155/2016/4732153

**Published:** 2016-11-03

**Authors:** Gulsum Uysal, Hakan Nazik, Nefise Tanridan Okçu, Sevtap Seyfettinoglu, Halil Kazgan

**Affiliations:** Department of Obstetrics and Gynecology, Adana Numune Education and Research Hospital, Adana, Turkey

## Abstract

Intrauterine devices (IUDs) remain highly effective reversible family planning methods in developing countries. We aimed to report one of the complications of extrauterine and intrauterine devices. A 44-year-old woman was admitted to our hospital with mislocated intrauterine device and abnormal uterine bleeding. Extrauterine IUD device was proven by ultrasound and X-ray. She had normal blood test count with a negative pregnancy test. There are several cases of complications with intrauterine devices, but this is the first case report about an extrauterine IUD embedded by inflame enlarged appendix presenting with abnormal uterine bleeding. Although intrauterine devices are a common safe method for contraception, there is no risk-free insertion even with advanced ultrasounds. A regular self-examination should be taught to the patients and ultrasonography should be performed in the follow-up of the patients especially for inserted devices during lactation period. Extrauterine IUDs can be successfully removed by laparotomy.

## 1. Case Report

**Figure 1:**
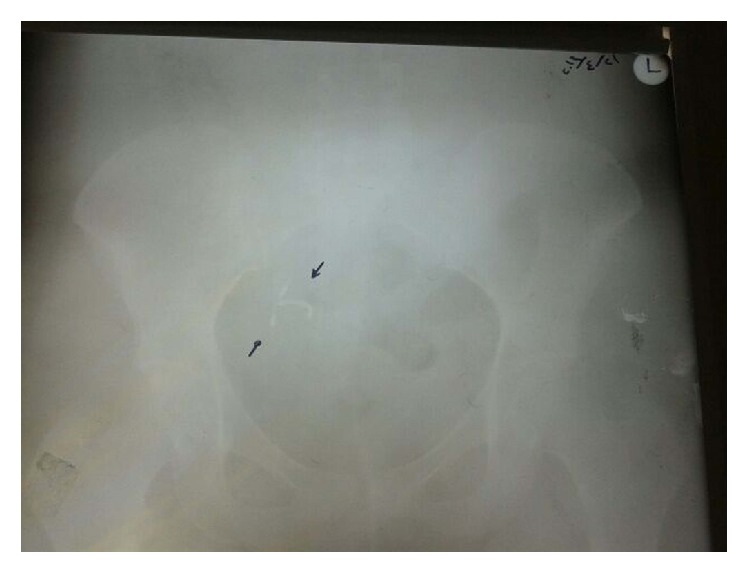
Abdomen X-ray of the patient. Copper T device was in the upper right side of the pelvis.

A 44-year-old Gravida 3, Parity 2, Abortion 0, Live 2, Syrian woman presented with abnormal uterine bleeding and transient pelvic cramps. She had no other complaints and had soft abdomen, with normal vital findings (afebrile, 120/80 mmHg). She had a Copper T A380 IUD, inserted one year ago during lactation. Her last birth was normal vaginal delivery two years ago and she was still breastfeeding. In her perspeculum examination, there was no thread of IUD. Following ultrasonography examination, a mislocated IUD was seen. The position of the uterus was anteverted (cervix angles forward) anteflexed (body is flexed forward) with endometrial thickness of 8 millimeters. An X-ray of her abdomen was performed and finally Copper T was revealed in the right side of her abdomen (Figure 1).

In laboratory, she had normal blood test count (hemoglobin count of 12.2 g/dL, white blood cell count of 7.9 × 10^3^/*μ*L, platelet count of 452 × 10^3^/*μ*L, and thyroid stimulating hormone of 1.7 uIU/mL) with a negative pregnancy test (beta-hCG (total hCG) < 0.1 mIU/mL).

**Figure 2:**
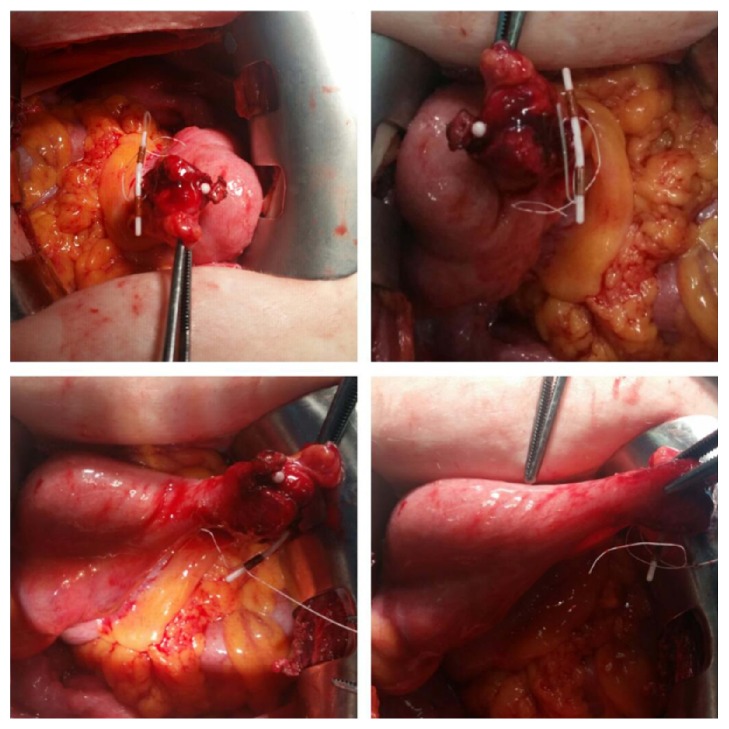
IUD was partly buried in right adnexa and was embedded by inflame enlarged appendix.

After obtaining her written informed consent, laparotomic operation was planned. Laparotomy with a Pfannenstiel incision was performed under general anesthesia. Intraoperatively, the IUD was found to be partly buried in right adnexa and was firmly attached to right tube and ovary. Scar tissue was not seen in the uterine fundus or corpus. With blunt and sharp dissections, tuba and ovary were remained unattached without any harm or lesions. At the bottom of attached tissue, IUD was also embedded by inflame enlarged appendix (Figure 2). IUD was embedded in the appendix and appendectomy was performed. There was no intraoperative complication.

The patient did well after operation and was discharged on the second postoperative day. Histopathological examination revealed chronic appendicitis, total peritonitis, edema, and fibrosis.

## 2. Discussion

Intrauterine devices (IUDs) remain highly effective, reversible family planning methods in developing countries. There are various complications of intrauterine devices such as bleeding, perforation, and migration to adjacent organs or omentum [1]. One of the most serious complications is uterine perforation (0.2–3.6/1000) especially inserted during lactation [1]. Although perforation of the uterus by an IUD is rare, migration to the appendix is extremely rare. Here, we report a case of migration of an IUD to the appendix.

IUDs have been used worldwide since 1965 [2]. There are two main types of IUDs: nonhormonal metallic copper releasing IUDs and hormonal levonorgestrel releasing IUDs (LNG-IUD) [3]. It is still the most common family planning method in our country because of the low cost, long lasting protection, and lack of systemic side effects [3]. However, there are several complications such as abdominal pain, menorrhagia, infection, and uterus perforation [2]. Misplaced IUDs have been presented and described from several organs such as the intestinal tract [4] and urinary bladder [5]. They may also be founded buried in the omentum [6]. They may lead to perforation of the uterus and migrate to neighbouring organs such as rectum and appendix [7], as this case report has suggested. Uterine or neighbouring organ perforations can occur during insertion or later. It was showed that in most of the cases the mislocated IUDs were inserted within 1 year after deliveries [1, 8].

The mechanism of migration is thought to be traumatic insertion during the procedure itself and with the help of chronic inflammatory reaction causing erosion of the device through the uterine wall. The incidence of uterine perforation is reported in the range of 0.2–3.6 per 1000 [8]. The incidence is accelerated by several factors, which include timing of insertion such as lactation period, the parity, experience of the operator, and the position of the uterus. Late symptoms are thought to be secondary migration with inflammatory process. One of the reasons of migration to an adjacent organ may occur through movements of omentum. Also enlarging uterus in unintended pregnancies and tubal ectopic pregnancy may cause migration [2, 8, 9].

In our case, IUD was performed one year ago in a local clinic during lactation period. During lactation period, both endometrial atrophy due to hypoestrogenic stage and accelerated involution of the uterus can lead to uterine perforation easily [7]. Therefore, it is suggested to use copper intrauterine devices within less than 48 hours or more than 4 weeks of time after delivery in postpartum and breastfeeding or not breastfeeding period [10].

The presence of copper in the abdominal cavity may lead to peritonitis and adhesions causing fibrosis. We suggest that mislocated IUD in the abdominal cavity in asymptomatic patients should be removed urgently. Also in our case living as a refugee may cause inflammation process due to public conditions and it may accelerate abdominal symptoms but she ignored her periodic pain until abnormal uterine bleeding. She had copper IUD, too.

Kaplanoğlu et al. retrospectively evaluated the data of 21 extrauterine IUD cases in their clinic in two years' time [9]. They reported that a total of 14 copper and seven LNG-IUDs were used and 71,4% of the patients were in the lactation period. Laparoscopy was performed in 14 patients. Laparotomy was performed in 7 patients because of severe adhesions. Most of the extrauterine IUD cases were located in the Douglas pouch while others were found in retroperitoneum, near the ovary, and in omentum with one in near the uterine artery. None of them were located in appendix. Mislocated IUDs in omentum and retroperitoneum were converted to laparotomy because of dense adhesions [9].

An ultrasound screening is usually recommended as first line examination to control the location of IUD [1]. The strings of device cannot be visualised during ultrasound examination in misplaced lost IUDs. Plain anteroposterior abdominal radiography is usually performed secondly to verify the presence of an IUD in the pelvis. Once it is found, secondary ultrasound examination of choice can be done to decide or detect the neighbourhood of the uterus as intestinal tract or urinary system. Computerized tomography can be used in complex cases where visceral involvement or surgical difficulty is suspected [1].

The treatment of mislocated IUDs is surgical and can be performed either laparoscopically or laparotomically [11]. Because of minimally invasive technique, the preferred surgical treatment is laparoscopy. The option of treatment depends on the degree of perforation and clinical symptoms (peritoneal sepsis, acute abdominal pain, and intestinal obstruction). It should be selected for every patient individually. Moreover, surgical experience is important for the operator. In our case, because of the suspicion of adhesions with bowel involvement mostly like ceacum (as it was located in the right upper pelvic area in the abdominal radiography), we preferred laparotomy. On the other hand, laparoscopic removal of the mislocated IUD could be difficult due to intraabdominal adhesions with possible bowel injury and may cause harm to intestines.

## 3. Conclusion

An IUD is an essential safe method for contraception. A regular self-examination should be taught to the patients and caregivers should be awake for early and late symptoms of mislocated devices. Ultrasonography should be performed in the follow-up of the patients after IUD insertion. In addition, during postpartum period (breastfeeding or not breastfeeding) IUD methods are recommended more than 4 weeks after delivery. Finally, IUD can be successfully removed by laparotomy.

## References

[1] Caliskan E., Öztürk N., Dilbaz B. Ö., Dilbaz S. (2003). Analysis of risk factors associated with uterine perforation by intrauterine devices. *European Journal of Contraception and Reproductive Health Care*.

[2] Zakin D., Stern W. Z., Rosenblatt R. (1981). Complete and partial uterine perforation and embedding following insertion of intrauterine devices. *Obstetrical and Gynecological Survey*.

[3] Kaislasuo J., Suhonen S., Gissler M., Lähteenmäki P., Heikinheimo O. (2013). Uterine perforation caused by intrauterine devices: clinical course and treatment. *Human Reproduction*.

[4] Key T. C., Kreutner A. K. (1980). Gastrointestinal complications of modern intrauterine devices. *Obstetrics and Gynecology*.

[5] Demirci D., Ekmekcioglu O., Demirtas A., Gulmez I. (2003). Big bladder stones around an intravesical migrated intrauterine device. *International Urology and Nephrology*.

[6] Kriplani A., Garg P., Sharma M., Agarwal N. (2005). Laparoscopic removal of extrauterine IUCD using fluoroscopy guidance: a case report. *Journal of Gynecologic Surgery*.

[7] Ohana E., Sheiner E., Leron E., Mazor M. (2000). Appendix perforation by an intrauterine contraceptive device. *European Journal of Obstetrics Gynecology and Reproductive Biology*.

[8] Johri V., Vyas K. C. (2013). Misplaced intrauterine contraceptive devices: common errors; uncommon complications. *Journal of Clinical and Diagnostic Research*.

[9] Kaplanoğlu M., Bülbül M., Yüce T., Kaplanoğlu D., Aban M. (2015). Mislocated extrauterine intrauterine devices: diagnosis and surgical management. *Journal of the Turkish German Gynecology Association*.

[10] (2015). *Medical Eligibility Criteria for Contraceptive Use*.

[11] Ozgun M. T., Batukan C., Serin I. S., Ozcelik B., Basbug M., Dolanbay M. (2007). Surgical management of intra-abdominal mislocated intrauterine devices. *Contraception*.

